# Martius flap closure of rectovaginal fistula in a 12-year-old girl with Crohn’s disease

**DOI:** 10.1093/jscr/rjaf768

**Published:** 2025-09-29

**Authors:** Hadjar Nassiri, Souha Qarouach, Jaouad Bouljrouf, Monim Ochan, Mounir Kisra

**Affiliations:** Faculté de Medecine et de Pharmacie de Rabat, Université Mohammed V de Rabat, BP 6203 – Rabat-Instituts, Avenue Mohammed Belarbi El Alaoui, 10100 Rabat, Morocco; Children's Hospital, Ibn Sina University Hospital Center, Avenue Ibn Rochd, Quartier Souissi, 10100 Rabat, Morocco; Laboratory of Life and Health Sciences, Faculty of Medicine and Pharmacy of Tangier, Abdelmalek Essaadi University, B.P. 365 Gzenaya, KM 15, Route de Rabat – 90080 Tetouan, Morocco; Faculté de Medecine et de Pharmacie de Rabat, Université Mohammed V de Rabat, BP 6203 – Rabat-Instituts, Avenue Mohammed Belarbi El Alaoui, 10100 Rabat, Morocco; Children's Hospital, Ibn Sina University Hospital Center, Avenue Ibn Rochd, Quartier Souissi, 10100 Rabat, Morocco; Faculté de Medecine et de Pharmacie de Rabat, Université Mohammed V de Rabat, BP 6203 – Rabat-Instituts, Avenue Mohammed Belarbi El Alaoui, 10100 Rabat, Morocco; Children's Hospital, Ibn Sina University Hospital Center, Avenue Ibn Rochd, Quartier Souissi, 10100 Rabat, Morocco; Faculté de Medecine et de Pharmacie de Rabat, Université Mohammed V de Rabat, BP 6203 – Rabat-Instituts, Avenue Mohammed Belarbi El Alaoui, 10100 Rabat, Morocco; Children's Hospital, Ibn Sina University Hospital Center, Avenue Ibn Rochd, Quartier Souissi, 10100 Rabat, Morocco; Faculté de Medecine et de Pharmacie de Rabat, Université Mohammed V de Rabat, BP 6203 – Rabat-Instituts, Avenue Mohammed Belarbi El Alaoui, 10100 Rabat, Morocco; Children's Hospital, Ibn Sina University Hospital Center, Avenue Ibn Rochd, Quartier Souissi, 10100 Rabat, Morocco

**Keywords:** Martius flap, Crohn’s disease, rectovaginal fistula, surgical management, case report

## Abstract

Rectovaginal fistulas (RVFs) are rare but severe complications of Crohn’s disease (CD), particularly uncommon in the pediatric population. They present with debilitating symptoms and are notoriously difficult to manage. We report the case of a 12-year-old girl with CD who presented with vaginal passage of stool and gas. Examination under anesthesia revealed a low RVF, and rectoscopy demonstrated distal rectal incomplete stenosis. Given the severity of inflammation and poor local healing conditions, a protective ileostomy was performed prior to definitive surgical repair. Once the perineal environment stabilized, the fistula was successfully treated using a Martius flap—a technique involving transposition of the bulbocavernosus muscle into the rectovaginal septum. Postoperative recovery was uneventful with no recurrence. This case highlights the importance of a multidisciplinary, staged approach in managing complex RVFs in pediatric CD and supports the use of Martius flap repair as a viable sphincter-preserving technique in selected cases.

## Introduction

Rectovaginal fistulas (RVFs) are one of the most infrequent yet life-altering complications of Crohn’s disease (CD) [1]. Though rare—occurring only in up to 10% of female patients with CD [2]—RVFs can cause debilitating symptoms, ranging from functional as the passage of stool or gas through the vagina, persistent discharge, dyspareunia, pelvic pain, to a psychological impact and social embarrassment [1, 2].

While perianal fistulas develop in ~25% of patients over a 20-year course of CD [2], RVFs are far less common and exceptional in children, where clinical experience and treatment data remain extremely limited. The multiple surgical options and lack of consensus among experts highlight the complexity and complications encountered to treat the disease surgically [3].

The pathogenesis often involves deep ulceration and transmural inflammation extending from the anorectum into the vaginal wall [4]. RVFs are categorized as complex fistulas under the American Gastroenterological Association classification [5], their management is particularly challenging due to high recurrence rates and impaired healing [6].

Initial evaluation typically includes physical examination, imaging, and examination under anesthesia (EUA), which remains crucial for assessing fistula anatomy and sphincter integrity [7]. Medical treatment, including anti-TNF-α agents, offers limited efficacy in RVFs compared to other perianal fistulas, likely due to poor tissue penetration and local anatomical factors [8].

When symptoms persist despite medical therapy, surgical intervention becomes necessary. However, reported success rates range widely—from 16% to 80%—depending on surgical technique, fistula complexity, and patient-specific factors [9]. Among the various approaches, The Martius flap, that was first described by Dr Heinrich Martius, in 1928 for the management of rectourethral fistulae [10], using a well-vascularized labial fat or bulbocavernosus muscle pedicle, is employed for complex or recurrent fistulas to reinforce the vaginal and rectal walls [11].

Here, we report the case of a high-risk patient with Crohn’s-related RVF who had successful healing following Martius flap repair, emphasizing the importance of tailored, multidisciplinary management in this rare and complex condition.

## Case report

**Figure 1:**
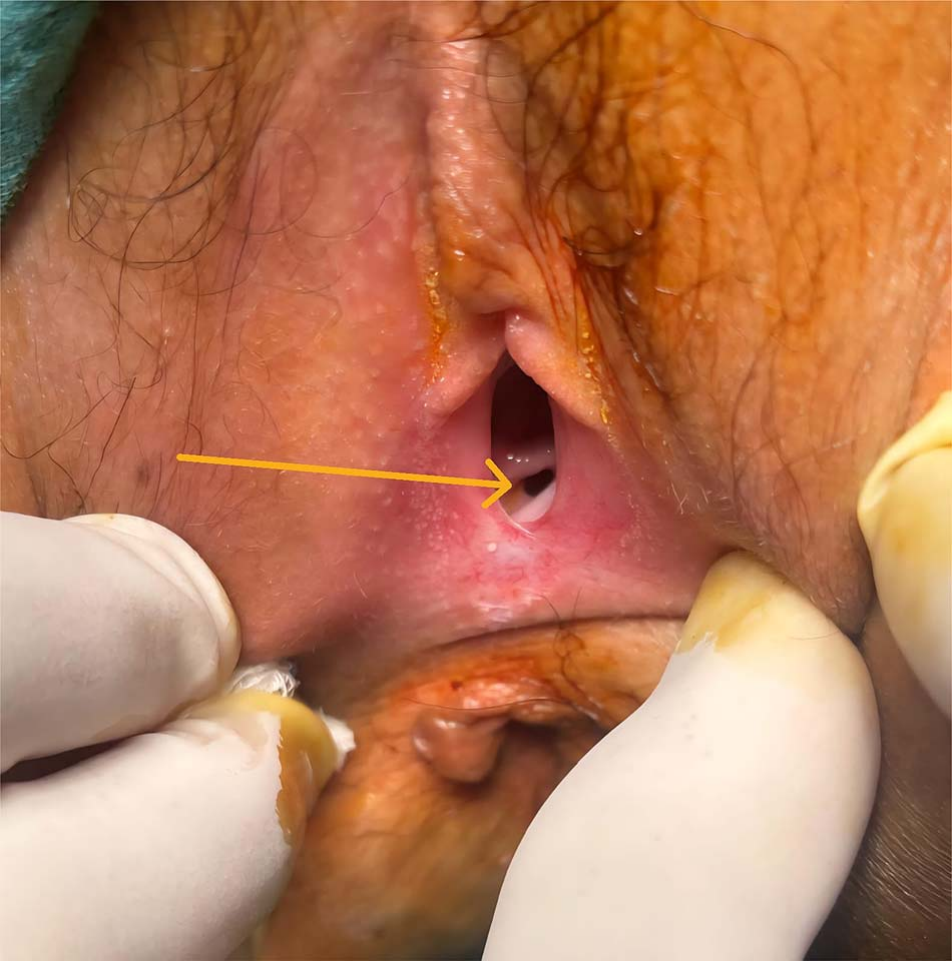
Rectovaginal fistula visible at clinical examination under anesthesia (arrow pointing the vaginal orifice of the fistula).

**Figure 2:**
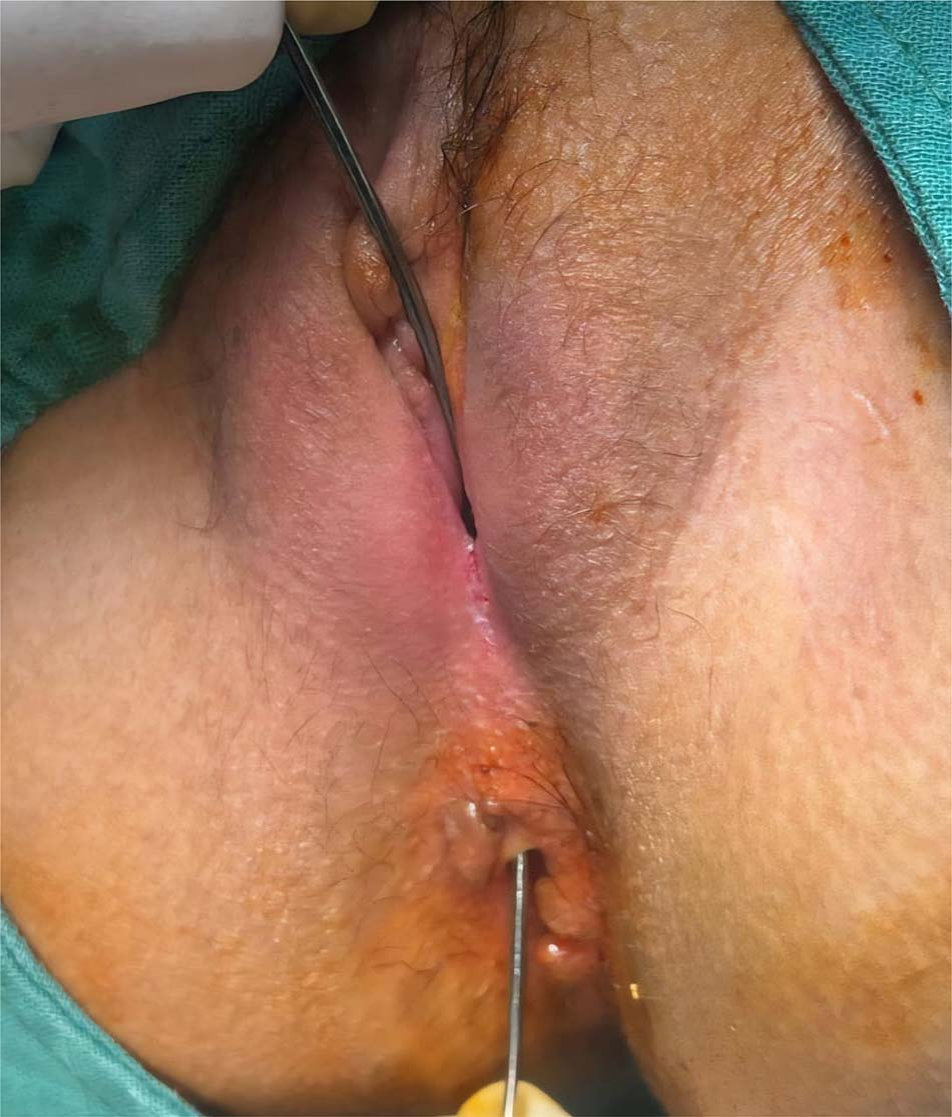
Introduction of a stylet through the vaginal orifice and advanced into the fistulous tract, demonstrated direct communication with the rectum, confirming the diagnosis.

A 12-year-old girl with a known history of CD presented with the passage of gas and stool through the vagina, consistent with a RVF. EUA revealed a distal RVF, clearly visible and easily probed (Figs 1 and 2). Rectoscopy demonstrated significant distal rectal incomplete stenosis, suggesting chronic inflammation and fibrotic narrowing.

**Figure 3:**
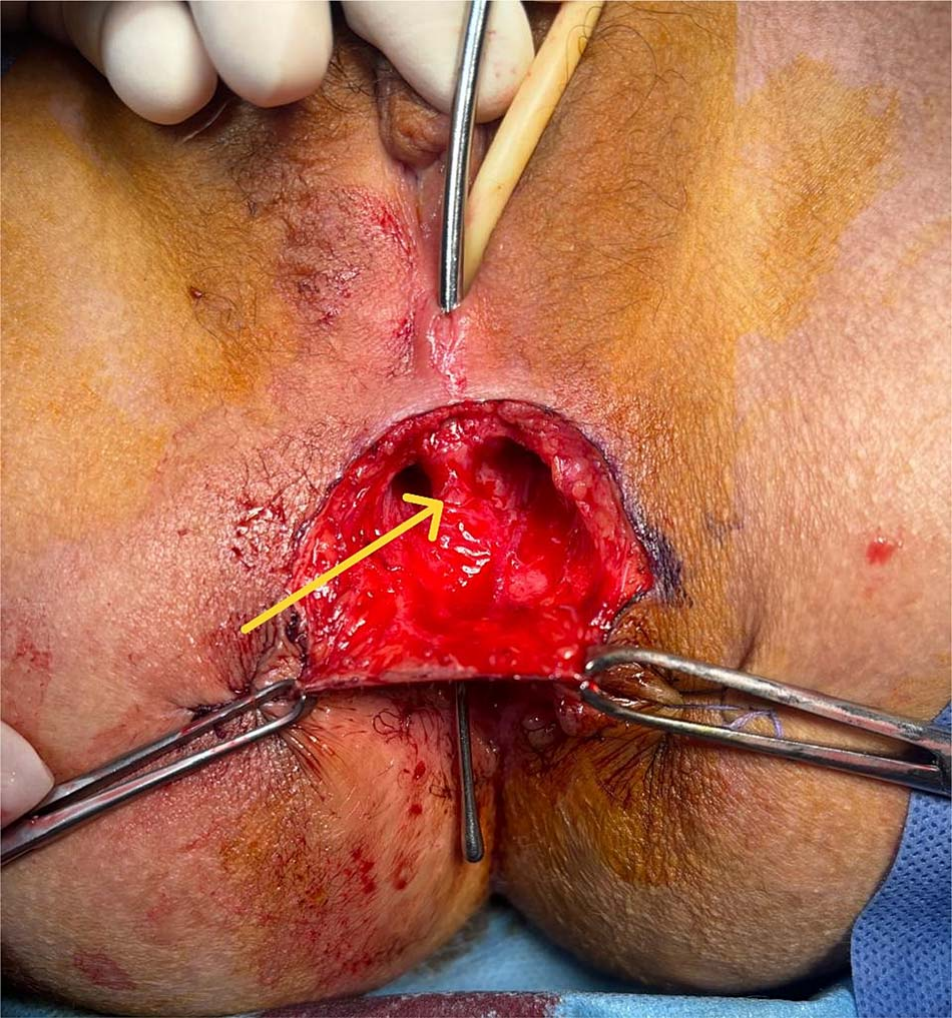
Perioperative image showing the fistulous tract after dissection of the subcutaneous tissue.

**Figure 4:**
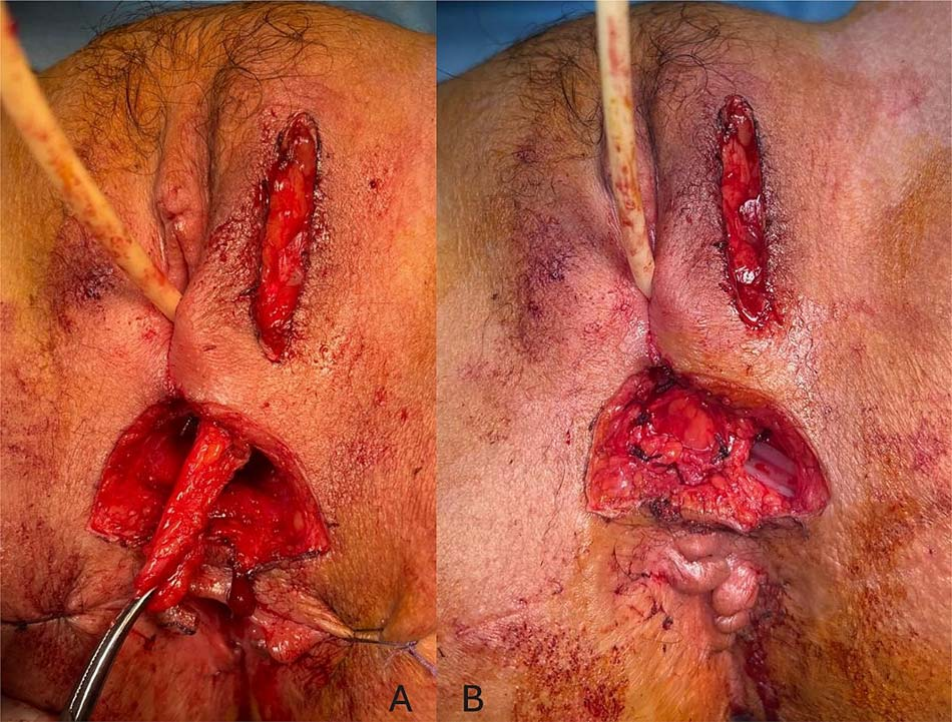
Perioperative images showing the dissection of the Martius flap (A) and its placement between the vagina and the rectum (B).

Given the unfavorable conditions for healing—including active perianal disease, stenosis, and poor tissue quality—a protective ileostomy was performed to divert fecal flow and optimize the local environment prior to definitive surgical repair. Once the inflammation was controlled and local conditions improved, a Martius flap procedure was undertaken. The most crucial steps of the surgical procedure included dissection of the fistula (Fig. 3) and closure of the rectal wall, then we proceeded to the preparation of a vascularized Martius Flap (Fig. 4) that was positioned between the vagina and the rectum. A redon drain was placed and kept for 48 h. The postoperative course was uneventful, there was no recurrence of the fistula at 3 and 6 months’ follow-ups.

## Discussion

RVFs in CD remain one of the most complex and frustrating challenges in colorectal surgery, not only due to their anatomical complexity but also because of their profound physical and psychosocial impact [1].

Although medical therapy can help reduce local inflammation, it often fails to achieve fistula healing, particularly in RVFs [8]. Surgical intervention is therefore typically required. However, complete and sustained closure frequently necessitates multiple procedures, as RVFs are known to require repeated interventions before durable healing is achieved [12].

This case highlights the successful use of a staged approach involving fecal diversion followed by Martius flap interposition for RVF in a 12-year-old girl with CD. The role of fecal diversion in surgical success remains controversial. Some authors report that diverting the fecal stream offers no statistically significant benefit to fistula closure rates [13]. In a review of 12 studies involving Martius flap procedures, only four were performed without a protective stoma; the rest employed either a loop colostomy or ileostomy [11]. However, even with a stoma, some fecal matter may still pass through the surgical site. Nonetheless, many authors agree that a diverting stoma can be instrumental in controlling sepsis and optimizing local tissue conditions prior to definitive repair [14].

In our case, the decision to perform a diverting ileostomy was based on active perianal disease and rectal stenosis, both of which are known to impair healing outcomes. This strategy aligns with published guidance, which emphasizes that controlling infection and inflammation should be the initial therapeutic goal in RVF management [15].

Surgical techniques for RVF closure in CD include advancement flaps, bioprosthetic implants, and muscle transpositions. Among these, the Martius flap offers a well-vascularized interpositional barrier and is especially useful in patients with compromised local tissue due to inflammation, scarring, or previous radiation [11]. Göttgens *et al*. report that Martius flap success rates range from 65% to 100%, with lower outcomes (65%–70%) in radiation-induced or Crohn’s-related RVFs [16]. These findings are in line with results from Sapci *et al*., who observed a 70% success rate in a tertiary referral center [17].

Long-term outcomes, however, remain sobering. In one of the largest studies on CD-related RVFs with extended follow-up, Tracanelli *et al*. reported a strict remission rate of only 22% and a high permanent stoma rate of 41% [3]. These data underscore the persistent challenges in managing RVFs and support the need for individualized, multidisciplinary approaches.

Our case adds to the limited body of literature on pediatric RVF in CD and demonstrates that Martius flap repair, when performed in a well-staged manner following appropriate diversion and local optimization, can lead to a successful and durable outcome—even in a high-risk, pediatric patient.

## Conclusion

This case highlights the complexity of managing CD-related RVF in pediatric patients and supports the value of a staged, individualized approach. Early diagnostic evaluation, thoughtful surgical planning, and the use of well-established yet adaptable techniques like the Martius flap can together yield excellent outcomes, even in high-risk scenarios. Further data are needed to establish consensus on optimal treatment pathways, particularly in younger populations.
